# Supplementary material for: Magnitude of workplace violence and associated factors among healthcare professionals in East Africa: A systematic review and meta-analysis
Source: PLoS One. 2025 Sep 19;20(9):e0332415. doi: 10.1371/journal.pone.0332415 (PMC12448341; doi:10.1371/journal.pone.0332415)
Supplement: S2 File — (DOCX) [file pone.0332415.s002.docx]

**Supplemental 2 file**: Searching approach

The adopted CoCoPoP format was used to retrieve relevant studies. The CoCoPoP consists of

Condition (Co), context (Co), and population (Pop) as described below.

**A. Condition**: prevalence of workplace violence.

**B. Context**: East African countries.

**C. Populations**: All healthcare professionals.

Using the above CoCoPoP, we constructed the following review questions which focused on retrieving relevant studies.

1. What is the prevalence of workplace violence among healthcare professionals in East Africa?

2. What are the factors affecting workplace violence among healthcare professionals in East Africa?

The primary studies were subsequently retrieved from the Google Scholar, PubMed, and Web of Science databases using the following keyword and MeSH terms: "workplace violence" OR "threaten" OR "aggression" AND "prevalence" OR "magnitude" AND "associated factors" OR "predictors" AND "healthcare professionals" OR "nurses" OR "doctors" OR "pharmacists" AND "East African countries". The search string was developed using ʺANDʺ and ʺORʺ Boolean operators.

| Databases | Key search terms or phrases |
| --- | --- |
| Google Scholar | "Workplace violence" OR "threaten" OR "aggression" AND "prevalence" OR "magnitude" AND "associated factors" OR "predictors" AND "healthcare professionals" OR "nurses" OR "doctors" OR "pharmacists" AND "East African countries" |
| Total articles | 510 |
| Manual Search and university repository | Prevalence of workplace violence and associated factors among healthcare professionals in East Africa |
| Total articles | 23 |
| PubMed | "Workplace violence" OR "threaten" AND "prevalence" OR "magnitude" AND "associated factors" OR "predictors" AND "healthcare professionals" OR "nurses" OR "doctors" OR "pharmacists" AND "East African countries" |
| Total articles | 510 |
| Web of Science | "Workplace violence" OR "aggression" AND "prevalence" AND "associated factors" OR "predictors" AND "healthcare professionals" OR "nurses" AND "East African countries" |
| Total Articles | 200 |
| Total retrieved articles from all databases | 1,243 |
