# Supplementary material for: Magnitude of workplace violence and associated factors among healthcare professionals in East Africa: A systematic review and meta-analysis
Source: PLoS One. 2025 Sep 19;20(9):e0332415. doi: 10.1371/journal.pone.0332415 (PMC12448341; doi:10.1371/journal.pone.0332415)
Supplement: S3 File — (DOCX) [file pone.0332415.s003.docx]

**Supplemental 3 file: Titles and outcome measurement tools of the included studies**

| Author (Year) | Title of included studies | Outcome measurement tools | Reported magnitude of workplace violence |
| --- | --- | --- | --- |
| Bekalu et al (2023) | Prevalence of Workplace Violence and Associated Factors Against Nurses Working in Public Hospitals in Northeastern Ethiopia | A self-administered structured questionnaire | 56 |
| W/Senbet et al(2022) | Sexual Harassment and Associated Factors Among Female Nurses: The Case of Addis Ababa Public Hospitals | A semi-structured questionnaire | N/A |
| Abeya et al (2018) | Workplace Violence among Health Care Workers and Associated factors in Public Hospitals of East Shoa Zone, Oromia Region, Ethiopia | A self-administered questionnaire | 70.2 |
| Legesse et al (2022) | Workplace violence and its associated factors among nurses working in public hospitals of eastern Ethiopia: a cross-sectional study | A self-administered questionnaire | 64 |
| Anose et al (2024) | Workplace violence and its associated factors among nurses working in university teaching hospitals in Southern Ethiopia: a mixed approach | A self-administered questionnaire and interview for qualitative part | 61.3 |
| Likissa et al (2014) | Assessment of the Prevalence and Predictors of Workplace Violence Against Nurses Working in Referral Hospitals of Oromia Regional State, Ethiopia | A self-administered structured questionnaires and interview for qualitative part | 82.8 |
| Alemu et al (2024) | Factors Associated with Workplace Violence among Nurses in Public Hospitals in Addis Ababa, Ethiopia in 2023: A Cross-Sectional Study | A self-administered questionnaire, | 67.5 |
| Abate et al (2019) | Prevalence and Associated Factors of Violence against Hospital Staff at Amanuel Mental Specialized Hospital in Addis Ababa, Ethiopia | A self-administered questionnaire | N/A |
| Dagnaw et al (2021) | Working in labor and delivery unit increases the odds of work place violence in Amhara region referral hospitals: Cross-sectional study | A self-administered structured | 44.5 |
| Tiruneh et al (2016) | Prevalence of workplace violence in Northwest Ethiopia: a multivariate analysis | A self-administered questionnaire | 26.7 |
| Mekonen et al (2020) | PREVALENCE OF WORKING PLACE VIOLENCE AMONG HEALTH CARE EMPLOYEES A CASE STUDY OF DESSIE REFERRAL HOSPITAL OF ETHIOPIA | A self-administered questionnaire | N/A |
| W/Hawaryat et (2020) | Prevalence of Workplace Violence and Associated Factors Against Nurses Working in Public Health Facilities in Southern Ethiopia | A self-administered and structured questionnaire | 43.1 |
| Wubneh et al (2023) | Prevalence and forms of workplace violence against nurses | A self-administered standardized questionnaire | 51.4 |
| Yenealem et al (2024) | Fear of violence and working department influences physical aggression level among nurses in northwest Ethiopia government health facilities | A standard questionnaire | 28.9 |
| Yenealem et al (2019) | Violence at work: determinants & prevalence among health care workers, northwest Ethiopia: an institutional based cross sectional study | A structured self-administered questionnaire | 58.2 |
| Tolera et al (2024) | Health service providers experience of psycho-emotional violence and associated factors among urban hospitals in Eastern Ethiopia | A structured questionnaire | 57.39 |
| Fute et al (2015) | High prevalence of workplace violence among nurses working at public health facilities in Southern Ethiopia | A structured questionnaire | 29.9 |
| Dagnaw et al (2022) | Sexual Harassment at the Workplace is Still a Hindrance among Midwives and Nurses Working in Northwestern Ethiopia Referral Hospitals: A Multicenter Cross-Sectional Study | A self-administered structured questionnaire | N/A |
| Kibunja et al (2021) | Prevalence and Effect of Workplace Violence against Emergency Nurses at a Tertiary Hospital in Kenya: A Cross-Sectional Study | A self-administered questionnaires | 77.8 |
| Atogo et al (2024) | Predictors of Work-Related Violence Against Nurses Working at a Tertiary Hospital in Kisumu, Kenya | A self-administered questionnaire | 70 |
| Musengamana et al(2022) | Workplace violence experience among nurses at a selected university teaching hospital in Rwanda | A structured, validated, and self-administered questionnaire | 58.5 |
| Newman et al (2011) | Workplace violence and gender discrimination in Rwanda’s health workforce: Increasing safety and gender equality | Researchers used a administered health worker survey, facility audits, key informant and health facility manager interviews and focus groups to collect data | 39 |
| Elhadi et al (2022) | Workplace violence against healthcare workers during the COVID-19 pandemic in Sudan: A cross-sectional study | A self-administered questionnaire | 78.3 |
| Elamin et al (2020) | Workplace Violence Against Doctors in Khartoum State, Sudan | A self-administered questionnaires t | 50 |
| Gaafar et al (2022) | Violence Against Healthcare Workers in Haj El-Sa Teaching Hospital, Sudan: A Cross-Sectional Study | A standardized closed-ended questionnaire | 54.9 |
