# Supplementary material for: Magnitude of workplace violence and associated factors among healthcare professionals in East Africa: A systematic review and meta-analysis
Source: PLoS One. 2025 Sep 19;20(9):e0332415. doi: 10.1371/journal.pone.0332415 (PMC12448341; doi:10.1371/journal.pone.0332415)
Supplement: Attachment — Submitted filename: Comments to the Authors.pdf [file pone.0332415.s004.pdf]

Manuscript Number: PONE-D-24-46926

Manuscript Title: Magnitude of workplace violence and associated factors among health professionals in East Africa. Systematic review and meta-analysis

Congratulations dear authors on your scholarly work based on a priori protocol registered in PROSPERO; you have brought an important study problem with good findings that have public health importance in optimizing health care professional safety and well-being in work place. However, there few methodological issues that I want you to address before considering the manuscript for publication.

Comments

Abstract

Introduction: This section needs to tell the reader about overview of burden of the problem, gap justification/ reason/aim of study.

Methods: better to remove the aim of the study from this part and add to introduction section. Please add sub group analysis, sensitivity analysis, study period etc…

Result: you need to reduce, make it short and precise (instead of writing associated variable and AOR separately write like (Emergency department (95%CI: 3.22, 5.39; I2 =93.71%, p = 0.00).Please write total study participants and included studies.

Conclusion: **"The magnitude of workplace violence in the region was high**" do you have benchmark to say high?

Your conclusions need to be based on your finding. I recommend you to place your Prospero registration here (Abstract)

Introduction (main): I feel it is well stated and organized except first write the full term before abbreviation, see your grammatical and language usages. In addition, you better to state the known factors/ variables, the attempted solution taken and planed strategies.

Methods: do you think that PIOC mnemonic is suitable for this review? Please revise it.

Please operationalize "workplace violence"

Result: Under "Characteristics of the included studies" be consistent write all in words or number (4, eighteen, 7)

It is better to narrate your result in intelligible, short and conclusive manner (revise it)

Discussion: I recommend you to revise your discussion section **critically**.

- Please use reference for your justification,

- Make it short and clear to transfer your message well to the reader
- There are some sentences which placed unwisely like “As primary articles revealed that……?
- The flow of idea is not smart
- Please use paragraph for distinct idea
- Generally it needs deep revision

Your conclusion should base your result

General comments

- ✓ There are several typological and grammar usage errors that need extensive proof reading for revisions.
- ✓ Don’t use the conjunction ‘**or’** to illustrate more, just one phrase or sentence is enough

Good luck!!
