# Supplementary material for: Magnitude of workplace violence and associated factors among healthcare professionals in East Africa: A systematic review and meta-analysis
Source: PLoS One. 2025 Sep 19;20(9):e0332415. doi: 10.1371/journal.pone.0332415 (PMC12448341; doi:10.1371/journal.pone.0332415)
Supplement: Attachment — Submitted filename: Response to reviewers.pdf [file pone.0332415.s006.pdf]

Point by point response for editor and reviewers

Journal Name: PLOS ONE, Manuscript Number: PONE-D-24-46926

To editor

We would like to express our gratitude for the opportunity to submit our revised manuscript, and invaluable comments and the time and effort he invested for our work

Point by point response to Reviewer 1

Dear reviewer, thank you for the time and efforts you have dedicated for reviewing and your valuable suggestions to improve our work

Reviewer #1: Manuscript Number: PONE-D-24-46926

Manuscript Title: Magnitude of workplace violence and associated factors among health professionals in East Africa. Systematic review and meta-analysis

Congratulations dear authors on your scholarly work based on a priori protocol registered in PROSPERO; you have brought an important study problem with good findings that have public health importance in optimizing health care professional safety and well-being in work place. However, there few methodological issues that I want you to address before considering the manuscript for publication.

Reviewers' questions or comments

Comments

Abstract

Introduction: This section needs to tell the reader about overview of burden of the problem, gap justification/ reason/aim of study.

Methods: better to remove the aim of the study from this part and add to introduction section.

Please add sub group analysis, sensitivity analysis, study period etc…

Result: you need to reduce, make it short and precise (instead of writing associated variable and AOR separately write like (Emergency department (95%CI: 3.22, 5.39; I2 =93.71%, p = 0.00).Please write total study participants and included studies.

Conclusion: "The magnitude of workplace violence in the region was high" do you have benchmark to say high?

Your conclusions need to be based on your finding. I recommend you to place your Prospero registration here (Abstract)

Authors' responses: Thank you for your comments and recommendations on improving structured abstract. **1. Introduction section**: we have revised the content of the introduction section of the abstract based on your comments. **2. Methods section**: in response to your recommendations, we have appropriately placed the study's aim and added subgroup analysis, sensitivity analysis, and the study period in this sections of the abstract. **3. Results section:** we have accept your suggestions to make the results section shorter and more precise, and we revised it accordingly. Additionally, we have added the total number of study participants and the number of included studies. 4. **Conclusion and use of benchmark:** Regarding the phrasing in the conclusion and the mention of a benchmark to define what is considered "high", we acknowledge that there is no established benchmark in this region. Therefore, we have revised the expression to reflect this and updated it in the revised manuscript. Finally, the Prospero registration number has been added to the abstract, as per your recommendation. Please see page 2, for all corrections.

Reviewers' questions or comments

Introduction (main): I feel it is well stated and organized except first write the full term before abbreviation, see your grammatical and language usages. In addition, you better to state the known factors/ variables, the attempted solution taken and planed strategies.

Authors' responses: Thank you for your insightful comments and recommendations regarding the use of abbreviations. We have taken your feedback into account and conducted a thorough review of abbreviations, grammar, and overall language usage in the revised manuscript. In addition, we have incorporated your suggestions regarding inclusion of variables/factors identified previously in the introduction section. Please see page 3, lines 20-21.

Reviewers' questions or comments

Methods: do you think that PIOC mnemonic is suitable for this review? Please revise it.

Please operationalize "workplace violence"

Authors' responses: Thank you for your constructive comments regarding PIOC and the need for operational definitions of workplace violence. We have taken your recommendations into account and changed PIOC to CoCoP design, both in response to your recommendations and to better align with the aim of the study in the revised manuscript. In addition, we have clearly operationalized the concept of workplace violence. Please see page 4 and 5 for CoCoP and page

6 for operational definition.

Reviewers' questions or comments

Result: Under "Characteristics of the included studies" be consistent write all in words or number (4, eighteen, 7)

It is better to narrate your result in intelligible, short and conclusive manner (revise it)

Authors' responses: Thank you for your comments regarding the inconsistency use of words and numbers in the descriptions of the characteristics of the included studies, as well as your suggestions to present the results in a more intelligible, concise, and conclusive manner. We have taken your feedback into account and consistently used words throughout this section. The manuscript has been carefully revised to improve clarity and readability. Please see page 7, lines 8-12.

Reviewers' questions or comments

Discussion: I recommend you to revise your discussion section critically.

• Please use reference for your justification,

• Make it short and clear to transfer your message well to the reader

• There are some sentences which placed unwisely like "As primary articles revealed that……?

• The flow of idea is not smart

• Please use paragraph for distinct idea

• Generally it needs deep revision

Authors' responses: Thank you for insightful comments and recommendations regarding discussion section. We have carefully considered your suggestions and incorporated them into the revised manuscript. Specifically, we have provided appropriate references for our justifications, used shorter sentences to enhance clarity, removed or revised unclear statements, improved the logical flow of ideas, and structured the text into distinct paragraphs to better separate different points. Please refer to the discussion section (9, 10 and 11) of the revised manuscript. We look forward to resolving any remaining issues.

Reviewers' questions or comments

Your conclusion should base your result

Authors' responses: Thank you for your constructive feedback. We have revised the conclusion section based on your recommendations in the updated manuscript. Please see page 11, lines 10-

18.

Reviewers' questions or comments

General comments

- There are several typological and grammar usage errors that need extensive proof reading for revisions.
- Don't use the conjunction 'or' to illustrate more, just one phrase or sentence is enough

Good luck!!

Authors' responses: Thank you for your thorough review and for recommending that we proofread the manuscript to improve its readability. We have carefully proofread the revised manuscript. In addition, we have removed unnecessary conjunctions to enhance clarity and flow. Please see the revised manuscript. We look forward to improving any typological and grammatical errors.

Point by point response to Reviewer 2

Dear reviewer, thank you for the time and efforts you have dedicated for reviewing and your valuable suggestions to improve our work.

Reviewer #2: Thank you for the opportunity to review this manuscript. This meta-analysis estimates the prevalence of life-long workplace violence among healthcare workers in East Africa, understanding workplace violence as having suffered at least once an aggression from a client or user. The meta-analysis is generally well perfomed and contains thorough analyses. I have one major comment which I hope will help improve this manuscript.

Reviewers' questions or comments

The meta-analysis 25 studies in its main analysis and obtains a robust estimation of the workplace violence prevalence. However, when it comes to analyzing moderator variables, the manuscript's approach is suboptimal in the sense that it does not take all of the possible studies. For instance, let me take the example of the analysis of sex as a potential variable. The manuscript only take those studies where men and women were compared and use the OR as a size effect. However, it is also possible to take the proportion of men and women in every study and perform a meta-regression, using the prevalence as the effect size, and thus taking all of the studies instead of only some of them. Same logic could be applied to age, and years of experience, and perhaps other moderating variables as well.

Authors' responses: Thank you for your insightful comments regarding conducting a meta-regression using the proportion of predictors (significantly associated factors) with workplace violence. We appreciate your constructive feedback on performing meta-regression in this manner when encountering high heterogeneity among the included studies. While we extracted data on the proportions of these independent variables, we did not conduct a meta-regressions, as there was no substantial variability among the included studies. We will use your recommendation to perform meta-regression for future works while facing variability.

Reviewers' questions or comments

The manuscript also mentions that the autohrs searched on databases like PubMed, Google Scholar, and Google. Is this the complete list of databases?

Authors' responses: Thank you for your questions regarding the amount of listed databases. In short, the response is "**No**". We retrieved articles from Web of Science, Google Scholar, PubMed, and Google manual search as well as online University repositories. Although we

searched multiple databases to retrieve relevant studies, none of them contained studies beyond those already included and mentioned in this manuscript. Please see page 6.

Reviewers' questions or comments

As a minor comment, regarding abbreviations and their use, it is customary that, the first time a term appears, both the expanded term and its abbreviation are mentioned, and in subsequent appearances only the abbreviation is used. I suggest the manuscript to abide to this custom. For instance, the first time the term appears is in the first sentence: there I would say "Workplace violence (WPV) is a violent...", and use only the abbreviation WPV afterwards. Same rule may be applied to the abbreviation AOR.

Authors' responses: Thank you for your constructive comments regarding the use of abbreviations and their explanations. We have taken your feedback into account and made the necessary corrections in the revised manuscript. Please refer to the complete revised manuscript for these corrections.

Reviewers' questions or comments

Last, in the last paragraph of the Methods, it is said that p-value "<0.05% was used". Do you mean a significance threshold of 0.05?

Authors' responses: Thank you for your comments regarding the description of p. value < 0.05%. In short, the answer to your question is "**No**". To determine the pooled significance of statistically associated factors with the outcome variable, we used a 95% confidence interval. We meant **"P-value of Q test < 0.05".** Please see page 6, line 21.

Point by point response to Reviewer 3

Dear reviewer, thank you for the time and efforts you have dedicated for reviewing and your valuable suggestions to improve our work.

Reviewers' questions or comments

Reviewer #3: In a meta-analysis, this paper deals with the questions of how high workplace violence is in East African countries and which predictors were found. It is a methodologically sound study. This and the results found are the strength of the work. However, I do see a some weaknesses in the work. Apart from a few points at the end, the introduction is good, but a chapter with related studies is missing. I am thinking in particular of systematic reviews and meta-analyses on the issues relating to East Africa, but also worldwide for comparison. For this reason, the discussion also suffers because there is no holistic reference to that existing literature. The methods section is complete in itself, but some more precise explanations are missing, and it could generally be more detailed. In my view, a very important question is why 70% of the studies were excluded due to unavailability before the full text review. It is very important to assess whether these could have an influence on the results.

I think this study is an important contribution to the documentation and prevention of workplace violence. I hope that you can revise this meta-analysis so that it can be published.

Authors' responses: Thank you for your constructive comments and for carefully reviewing the study selection process. When we refer to "**full texts**", we are specifically looking for studies that include comprehensive details on the **study population, study areas, and their outcomes to our research.** We apologize for any unclear phrasing or wording in the original manuscript, we have revised the expression accordingly in the revised manuscript. Therefore, by "**full texts unavailable"** in this context, we meant that one or more of the above parameters were not accessible. Please see the page 7, lines 1- 6.

Reviewers' questions or comments

General comments

• In chapter 3 (methods), 3.1 Data analysis follows 2.4 Data extraction. I suppose it should be 2.5 Data analysis.

You have no chapter 3. After chapter 3. Methods chapter 4. Results follows, and the 4. Meta-Analysis.

• Please, let proofread your paper. There as some grammatical errors and stylistic issues.

Authors' responses: Thank you for your constructive comments regarding the order of topics and sub-topics. We apologize for inconsistent use of these sections in the original manuscript. We addressed all the issues in the revised version. Please see the updated methods section of manuscript.

Reviewers' questions or comments

Abstract

The abstract is comprehensible and contains the relevant information.

• There are mentioned findings of a WHO study on WPV that are not mentioned in the introduction.

• I recommend to mention all databases searched not "like PubMed, …".

• Egger's test (not egger's test)

• AOR is used in the abstract. I would write out the abbreviation in the abstract and introduce the abbreviation at first use later in the paper.

• Do not reference to figures in the abstract ("fig.12").

• The second part of the results (in the abstract) "Emergency room AOR was …" is not written in complete sentences.

Authors' responses: Thank you for your insightful comments and suggestions, which have helped improve the clarity and readability of our manuscript.

1. Data in abstract but not in the introduction: we have checked it, but the mentioned figure (20%-30%) in the introduction section of the abstract also found in the main introduction section of the original manuscript**.** Please see page 3 lines 7 - 8.

2. Missed databases in the abstract: we have included all databases used to retrieve articles in the methods section of the abstract in the revised manuscript.

3. **Regarding capitalization, abbreviations, and fig 12 referenced in the abstract**: we have made appropriate corrections in the revised manuscript. Please see the abstract section of the revised manuscript.

Reviewers' questions or comments

Introduction

I see some issues in the last section of the introduction.

You write that WPV is "predominantly dangerous in Africa, especially in East African countries". Why is that the case. You should explain this.

"they are almost similar in resource, health staff and technology usage, infrastructures, health care practice and environment." -> similar to which countries.
You write that there is no comprehensive study on WPV in East Africa and that despite the importance there are few information. But then you write "It is therefore feasible to determine the magnitude workplace violence and its predictors in the region level."
That is not logic to me. If there are few studies, few information, how can you conduct a meta-analysis?

• There are phrases that are not easy to understand for me (e.g. "having almost similar healthcare institutions and challenges in the region of this")
• Introduce the abbreviation of workplace violence at it's first use; not further down in the introduction.
• "the rest employees" -> the other employees
• Several times in the paper you mention to conduct "a systematic review and a meta-analysis". I would only refer to meta-analysis. A meta-analysis is a kind of systematic review, but I would not mention both.
Authors' responses: Thank you for your comments and questions regarding the introduction section of the manuscript.
1. **Regarding why WPV dangerous in East Africa**: we have revised the expression regarding to workplace violence in East Africa.
2. Almost similar in resource, health staff, etc.: we used the term "**similar**" in this context to refer to all the countries included in this study (East African countries). We expressed the phrase in more understandable manner.
3. Regarding the expression of justification to conduct this study: by the expression '**no comprehensive study'** we meant that there is no pooled prevalence of WPV in the region, but this does not imply that there are no individual primary studies in each country. Please see page 4, lines 1 -7. We look forward to your suggestion on improving this expression of justification.
4. Regarding the expression of "rest employees" we corrected it by other sector employees.
5. Regarding unclear expressions and the use of abbreviations: we have taken your feedback into account and made appropriate corrections in the revised manuscript. In addition, we removed unnecessary repetitions of the terms "systematic review and meta-analysis" in the

revised manuscript. For more please see the revised manuscript.

Reviewers' questions or comments

Related works

I miss a chapter with related works, especially systematic reviews and meta-analyses. You might use these studies to show the actual state of the art and make first conclusions about WPV worldwide, in Africa and East Africa. Then you can bring them and their results in relation to the results of your paper in the discussion section. I listed some systematic reviews and meta-analysis further below.

Authors' responses: Thank you for your suggestions regarding the inclusion of more literature in the introduction section. We appreciate your efforts in listing relevant articles in relation to this work. We have incorporated these works into the introduction section of the revised manuscript, such as outcome variable (prevalence) and/or on the predictors of workplace violence. Please see page 3, lines 14 – 21.

Reviewers' questions or comments

Methods

In the methods section all necessary information is there, even if mostly in a rather short way. I recommend integrating the subchapters 4.1, 4.2 and 4.3 into the methods section.

But there are some points to clear.

• mObjective: "to determine the overall pooled magnitude of workplace violence and identify associated factors among healthcare professionals in East Africa"

But in the next phrase you write "to evaluate the category and predictors of workplace violence in this area". What do you mean with category? That seems to be a new element.

Authors' responses: Thank you for your constructive comments and recommendations regarding the integration of subchapters in the methods section. We also appreciate your observation regarding unnecessary uses of the term "**category**" in the objective of the study. Your thorough review of the phrases and wording throughout the manuscript is appreciated. We have removed it (**category**) and integrate subsections into methods section in the revised manuscript. Please see the methods section and the objective of the study in the revised manuscript.

Reviewers' questions or comments

• PRISMA: I would very brief explain what these guidelines are for.

• In subchapter 2.1, I do not find the description of the study design. There is the objective and the reference to the PRISMA guidelines and the register of systematic reviews.

• 2.1.1: The details of the search strategy are not clear to me. Did you mention all databases or only a part of them ("like PubMed, …")? Who was conducted the search for grey literature? In what databases? Did you find grey literature? How was "search complementary articles" done, by whom?

• Please explain shortly what the PIOC pursuing design is.

• "The explanation approach and quantity of rescued article to database has been given.» Where did you do this?

I recommend integrating the chapters below into the methods section, where there is the reference to fig. 1.

• Concerning data extraction, you write that you did extract "odds ratio of significantly associated factors". Did you leave out non-significant factors? That would bias results. I suppose I am misunderstanding something.

• "The associated factors of workplace violence were also the second interest of this review" correct: "The associated factors of workplace violence were the second interest of this meta-analysis."

• Egger's test (not egger's test)

• "I2 value of 0, 25%, 50 and 75%" -> "I2 value of 0%, 25%, 50% and 75%"

• Figure 1: What are other reasons for exclusion before screening

I am a bit alarmed to read that 89 of 127 studies are excluded due to unavailability of a full text. What is the unavailability? Are they not open source? Did you find only a reference but no functioning link or they seem not to exist? This seems important to me.

Whenever possible you should select these studies for full text review. If this is not possible you should estimate with the existing information (title, abstract) if these papers would change your findings.

Authors' responses: Thank you for your constructive comments and questions regarding missed descriptions, databases, data extractor names, explanations about PIOC design, the place of retrieved articles, the effect sizes (odds ratios of significant vs non-significant factors),

capitalization, and missing of the percentage sign. We have taken your recommendations into account and made appropriate corrections in the revised manuscript.

Regarding PRISMA reporting checklists and registration protocol: we apologize for the absence of details in the original manuscript. We have attached all necessary files in the revised manuscript. Please see page 4 and supplemental 1 file.

Concerning the search strategies: we have revised the search approach and provided an example from one database in the revised manuscript. See page 5 and supplemental 2 file

Regarding the name of the reviewer who conducted the search: we provided their names in the revised manuscript.

Regarding the PIOC pursuing design: we have changed the search design from PIOC to CoCoP based on the recommendations from reviewers and its suitability to the aim of this study. The study focused on the context, condition, and population (CoCoP) of included studies rather than population, intervention, outcomes, and comparison (PIOC). Please see page 4 and 5.

Concerning the integration of subsection: we appreciate your recommendation to integrate these subsections into methods section. We have made all necessary corrections in the revised manuscript.

Regarding the extracted data of odds ratio of significantly associated factors: we extracted effect sizes such as prevalence of WPV and adjusted odds ratios for factors significantly associated with WPV, as clearly stated in the eligibility criteria of this study.

Concerning the place of the explanations of the approach and quantity of rescued studies: we apologize for the absence of attached file about searching approach and quantity of rescued articles in the original manuscript. We have provided supplemental file regarding these in the revised manuscript. **Please see supplemental 2 file**.

When we refer to "**full texts**", we are specifically looking for studies that provide data on the **study population, study areas, and their outcomes relevant to our research.** We apologize for any unclear phrasing or expressions in the original manuscript. We have revised the wording accordingly in the revised manuscript. Therefore, by "**full texts unavailable"** in this context, we meant that one or more of the above parameters were not accessible. Please see page 7, lines 3 - 5. We look forward to making any modifications regarding this work.

Reviewers' questions or comments

Results

As mentioned above I recommend integrating chapter 4.1 to 4.3 into the method section.

• 4.3: the first sentence is a repetition (methods section)

Meta-analysis

When placing the chapters 4.1-4.3 into to method section, I would rename chapter 4. Meta-analysis in 4. Results.

All in all, the results are presented clear and understandable. Even if an editorial question, I would reflect about the integration of the figures within the text. Thirteen figures are a lot and most of them might be rather large compared to their contained information.

• When writing about the analysed papers I would use the word studies (not articles).

Authors' responses: Thank you for your comments on the integration of figures within the text. In response to your recommendation, we have used a table to reduce the number of figures and have also presented the information in textual format. In addition, we have replaced the expression "**articles'** by "**studies"** in the revised manuscript. Please see 8, lines 25 – 31 and attached table 3 in the revised manuscript.

Reviewers' questions or comments

Discussion

Principally, the discussion is ok. But as there are only view related studies are cited in the introduction, I miss a broader discussion in relation to existing literature (especially systematic reviews and meta-analyses).

• I would integrate numbers (results of other studies) into the discussion, e.g. "which is higher than the findings of studies conducted in Nepal[10], in France[43].» I would add there the lower values of the referred studies.

• Alcohol and substance consumption. Reflection about cause and effect: Alcohol or substance consumption could be a consequence of WPV not a cause.

• Women face more WPV. What about sexual harassment? That's not WPV per se, evtl. one version of it. Could it be that this is one reason for the higher values.

• This finding is supported with the result of other studies[45]. I would mention that [45] is a meta-analysis; else it seems strange to mention other studies and refer only to one.

• "This insecurity feeling of professionals directly affect the healthcare service provided and the

outcome of institutions. .” -> Two points at the end of the sentence.

Authors’ responses: Thank you for your insightful comments and recommendations regarding the discussion section of the manuscript, particularly your suggestions on using systematic reviews and meta-analyses for comparisons, including reported effect sizes (numerical values), and reflecting on alcohol or substance uses as potential causes or consequences of WPV.

Regarding numerical values: we have taken your feedback into account and added appropriate data (studies results) to each citation in the revised manuscript. Please see page 9, lines 11 – 18.

Regarding the reflection on cause and effect: We appreciate your concerns about alcohol and substance use as a consequence of WPV. Indeed, alcohol and substance use can be a consequence of WPV, as affected healthcare professionals may turn to these substances as a coping mechanism to manage or forget the trauma. However, alcohol and substance use can also be a contributing factor to WPV, particularly when healthcare professionals consume them either at home or in the workplace, leading to impaired brain function. This can impair performance, increase the likelihood of errors, and potentially lead to conflicts with patients or their families.

Regarding sexual harassment issues: Sexual harassment, while sometimes treated as a distinct category, is considered as a form of one workplace violence in our study if it is occurred on female healthcare professionals at their workplace. Basically workplace violence among healthcare professionals classified into four based on the **perpetrator** and **context of the violence. Type I:** Criminal Intent, **2. Type II:** Patient or Client Violence**, 3. Type III:** Worker-on-Worker, **and 4. Type IV:** Personal Relationship Violence. **Types of Acts within WPV (regardless of category) such as Physical violence, Verbal abuse, Sexual harassment or assault, Psychological or emotional abuse, and Stalking or intimidation.**

Sexual harassment may be one reason why women often face greater challenges than men in the workplace.

We have addressed the issue of using the phrase “**other studies”** when only as single study was cited, the expression have been corrected in the revised manuscript.

Regarding double **dot** in the conclusion and recommendation section: we have removed the double **dot**. Please see the discussion and conclusion sections of the revised manuscript.

Point by point response to Reviewer 4

Dear reviewer, thank you for the time and efforts you have dedicated for reviewing and your valuable suggestions to improve our work.

Reviewer #4: Dear editor,

Thank you for providing me with the opportunity to review this interesting work. The authors present a systematic review and meta-analysis of the prevalence of workplace violence among healthcare workers in East Africa and of factors that moderate its prevalence. The topic is certainly of interest, but I have strong reservations against the paper in its present form. I address only the most pressing points for now. Further issues would need to be discussed after any revision.

Reviewers' questions or comments

1) The text is sometimes difficult to understand. I suggest the authors use ChatGPT or similar to improve the grammar and readability of their manuscript.

Authors' responses: Thank you for your insightful comments regarding the grammar and clarity of the text. We have taken your feedback into account and have carefully proofread the manuscript to improve its readability. Please see the revised version. We look forward to correcting any flaws.

Reviewers' questions or comments

2) Out of 127 publications selected for full text review, 89 (i.e., about 75%) could not be accessed. I fully understand that access to research papers is limited at some institutions. However, a review that misses about three quarters of the relevant literature is not very useful. I suggest that the authors look for a collaboration partner who can provide them with access to the missing papers or find some other means to access these publications.

Authors' responses: Thank you for your constructive comments and for carefully reviewing the study selection process. When we refer to "**full texts**", we are specifically looking for studies that provide data on the **study population, study areas, and their outcomes relevant to our research.** We apologize for any unclear phrasing or expressions in the original manuscript. We have revised the wording accordingly in the revised manuscript. Therefore, by "**full texts unavailable"** in this context, we meant that one or more of the above parameters were not accessible. Please see the page 7, lines 3 - 5.

Reviewers' questions or comments

3) It remains unclear how the prevalence of workplace violence was assessed across studies. It is therefore unclear if the results can be pooled or compared. Imagine two studies that find 50% workplace violence. One study asked: "Did you experience workplace violence throughout the past 12 months?". The other asked "Have you heard of a colleague who experienced workplace violence throughout their career?" It would be misleading to assume that both studies found the same result. Please provide information how the experience of workplace violence was assessed in each study.

Authors' responses: Thank you for your questions regarding the outcome assessment methods used in the included primary studies. In this review, we simply pooled the reported prevalence of workplace violence from each study but not compared. While there may be variations in study quality, we have extracted and pooled the reported prevalence data as presented in the original studies.

Reviewers' questions or comments

I wish the authors all the best for their project and hope that they further build on the substantial work they have undertaken so far.

Johannes Hönekopp

Authors' responses: Thank you for your wish and hope regarding to our project. We appreciate your comments, which have helped improve the clarity and readability of our manuscript. We look forward to any corrections on our work.
