# Supplementary material for: Magnitude of workplace violence and associated factors among healthcare professionals in East Africa: A systematic review and meta-analysis
Source: PLoS One. 2025 Sep 19;20(9):e0332415. doi: 10.1371/journal.pone.0332415 (PMC12448341; doi:10.1371/journal.pone.0332415)
Supplement: Attachment — Submitted filename: Response_to_reviewers_auresp_2.pdf [file pone.0332415.s007.pdf]

Point by point response for editor and reviewers

Journal Name: PLOS ONE, Manuscript Number: PONE-D-24-46926

To editor

We would like to express our sincere gratitude to the editor for the opportunity to resubmit our revised manuscript. We deeply appreciate the invaluable comments, thoughtful guidance, and the time and effort dedicated to improving the quality of our work.

Editors' questions or comments

The author is asked to reasonably explain the third reviewer's recommendation, especially since he listed four articles to refute the author's argument.

Authors' response: Thank you for providing the list of articles mentioned by reviewer. While the editor's comments indicate that we should respond to four (4) studies, we were able to identify only three (3) articles cited in the reviewer's text. This is a gentle reminder to clarify the discrepancy between the stated number of articles (4) and the actual number we found (3). We were unable to locate the fourth article in the text forwarded from Reviewer 3.

Point by point response to Reviewer 3

Dear reviewer, thank you for dedicating your time and effort to review our work. We sincerely appreciate your insightful comments and valuable suggestions, which have been instrumental in helping us improve the clarity and readability of the manuscript.

Reviewer #3: The authors have incorporated most of the reviewers' suggestions for improvement or explained why they have not done so. The paper has improved significantly since the revision and might be ready for publication with minor revisions if two points are clarified.
In all, three points remain unclear to me:

Reviewers' questions or comments

1. Proofreading is still required.

Examples: The first sentence in the introduction is still incorrect, or rather, an error has been inserted (WPV occur instead of WPV occurs). Interestingly, the same sentence is better formulated in the abstract (even before the revision). 'Towards the staff' (abstract) or 'toward the staffs' (introduction) may be correct, but I would write 'against employees'. There are other examples such as: 'the impact of predictors on outcome variable' (that is 'variables'?). In the abstract, the list of moderators is not integrated into a correct sentence ('... with the outcome variable. Emergency department ...').

All this makes reading somewhat more difficult and leads to misunderstandings.

Authors' response: Thank you for your constructive feedback. We appreciate your recommendation regarding the sentence clarity. We have taken your recommendation into account and proofread the manuscript. Kindly refer to page 2 and 3 for specific feedback.

Reviewers' questions or comments

2. Studies with no full text

I understand the explanation for the exclusion of studies lacking "full texts" (still written this way on page 5), but I am still not convinced. I apologize for being so suspicious and persistent here. I might be a question of language, or expression that I don't understand correctly. But I cannot exclude that your explanation is a way to save the paper for publication.

The sentence 'During full-text review, 89 studies with no accessible full text were removed' is a clear and easily understandable statement that says that 89 studies were not accessible. The new wording 'During full-text review, 89 studies were excluded due to the full-text articles did not report the outcome of interest'. This is a completely

different statement. You excluded the studies because they did not meet the inclusion criteria.

Authors' response: Thank you for your follow-up regarding your previous question. We greatly appreciate your insightful comments, which have helped improve the clarity and readability of the manuscript. Please accept our sincere apologies for the confusion caused by the wording. The revised wording was intend to clarify the meaning of our original expression, not to defend the paper. You are welcome to verify, through your own searching, the presence or absence of any eligible studies that were excluded due to inaccessible full-texts. We look forward to receiving any suggestions regarding to the expression of this exclusion.

Reviewers' questions or comments

3. Most studies from Ethiopia

Of the 25 studies analysed, 18 (72%) are from Ethiopia. I wonder whether there are really no studies on WPV from other East African countries such as Tanzania, Mozambique or Madagascar. In the revised version, this weakness is mentioned in the discussion: 'Therefore, based on the subgroup analysis, we can conclude that the source of the heterogeneity may be the inclusion of studies from Ethiopia, Rwanda and Sudan.' I understand the sentence, although it is not a clear statement: 'we can conclude that [...] may be'.

However, in my view, there are studies on the subject from other East African countries: e.g.

- Patricio et al. (2022): https://doi.org/10.1002/hpm.3506
- Shimoda et al. (2020): https://doi.org/10.1186/s12884-020-03256-5
- El Ghaziri et al. (2014): https://doi.org/10.1016/j.jana.2013.07.002

If it is indeed the case that most of the studies are from Ethiopia, then this requires a plausible explanation or in-depth discussion.

If studies were indeed excluded because they were not accessible, and if there are indeed further studies from other East African countries, then I would not publish the paper. It would be better to complete data collection, reanalyse, adapt the text and resubmit the paper.

I apologize again for my mistrust, and I recommend speaking with the editor.

Authors' response: Thank you again for your insightful feedback on our manuscript. We appreciate your effort and dedication in searching and listing related studies, even though they fall outside our inclusion criteria. Further details are provided below.

- **Patricio et al. (2022): https://doi.org/10.1002/hpm.3506,** which conducted in **Mozambique**. According to standard classification by the Africa Union, United Nation, and World Bank, **Mozambique** is considered part of **Southern Africa**. However, our review included only studies conducted in **East Africa**. Therefore, the study by Patricio et al. was excluded based on geographic criteria. Please refer to the study's setting for more details.
- **Shimoda et al. (2020): https://doi.org/10.1186/s12884-020-03256-5** conducted in Tanzania. The study assessed workplace violence perpetrated by nurses and midwives against women during childbirth. As the literature shows, violence typically involves two key participants: perpetrators, who commit the act, and victims, who suffer harm or the threat of harm. Accordingly, the study examined self-reported instances of violent acts committed by nurses and midwives during childbirth. In this context (Shimoda et al.), nurses and midwives are considered perpetrators, not victims.

Although Tanzania is part of our study area and healthcare professionals (**nurses and midwives**) constitute our target population, this systematic review includes only studies that report workplace violence **against healthcare professionals** by service seekers. **Therefore, the study by Shimoda et al. was excluded due to a mismatch in the target population, as it focused on violence committed by, rather than against, healthcare workers.** Kindly see the full document of the study.
- **El Ghaziri et al. (2014):** https://doi.org/10.1016/j.jana.2013.07.002 conducted their study in Sub-Saharan Africa, which broadly includes East, West, Central, and Southern regions. However, their sample included healthcare professionals from countries such as Nigeria, Zimbabwe, Zambia, and Togo —none of which are part of East Africa. Therefore, the study by El Ghaziri et al. was excluded due to differences in the study area's geographic focus. Please refer to the full document for a complete list of included countries.

Reviewers' questions or comments

If it is indeed the case that most of the studies are from Ethiopia, then this requires a plausible explanation or in-depth discussion.

Authors' response: Thank you for your question regarding the dominance of studies from Ethiopia. Indeed, a large proportion of the included studies were conducted in Ethiopia, and have acknowledged this in the limitation section of the manuscript based on previous comments by reviewers. This dominance may reflect both the research focus and the availability of data on the topic in the region, rather than a deliberate selection bias.

Reviewers' questions or comments

I understand the sentence, although it is not a clear statement: 'we can conclude that [...] may be'.

Authors' response: Thank you for your feedback regarding the ambiguity between expressions certainty (can conclude) and uncertainty (may be). We have taken your insightful feedback into account and revised the expression. Please see page 8, lines 19-24.

Point by point response to Reviewer 4

Dear reviewer, thank you for dedicating your time and effort to review our work. We sincerely appreciate your insightful comments and valuable suggestions, which have been instrumental in helping us improve the clarity and readability of the manuscript.

Reviewer #4: I find the manuscript much improved, and I like the clear focus on applied consequences in the discussion. A number of problems still need to be addressed, though.

Reviewers' questions or comments

1. P7, "13 studies were also removed due to study design differences". What does this mean? Is this related to the exclusion of studies due to poor quality mentioned earlier? Please clarify.

Authors' response: Thank you for your feedback regarding the need for clarity on the study design differences. We appreciate your effort and dedication in helping us improve the clarity of manuscript.

Regarding the 13 studies that were removed due to study design differences: This means they were excluded specifically because their study designs did not align with our predefined inclusion criteria, not due to poor quality. Importantly, this exclusion occurred prior to the quality assessment step, which was conducted only on studies that fully met the inclusion criteria.

Reviewers' questions or comments

2. Regarding the proportion of staff experiencing violence, you present a test of publication bias (p7). I don't think that the concept of publication bias applies here. Whichever proportion of violence a study finds, applying any test of statistical significance does not make sense. It is just a single estimate, and there is no benchmark against which it could be sensibly tested against. Consequently, there cannot be bias against statistically non-significant studies. I therefore suggest you drop the test of publication bias.

Authors' response: Thank you for your constructive comment regarding the test of publication bias. We really appreciate your suggestion and feedback on this test. In meta-analysis of proportion (prevalence) data, each study contributes a single effect estimate, which can be used to construct a funnel plot in STATA. Although each study reports only one estimate, a funnel plot can still be created by plotting the following:

X-axis (horizontal line): The effect size, typically the logit-transformed prevalence (proportion) from each study.

Y-axis (vertical line): A measure of precision, commonly the inverse of the standard error of the transformed estimate (prevalence).

This funnel plot is centered on the pooled estimate and includes 95% confidence intervals, allowing for visualization of the spread of effect sizes relative to their precision— even when based on a single estimate. In our study, the funnel plot used to assess publication bias was constructed according to this principle. The asymmetry observed in the funnel plot was confirmed by Egger's test.

Egger's test examines weather there is a systematic relationship between the effect sizes of the study and their standard errors (a proxy for study precision). Egger's test was conducted by regressing the standard normal deviate (i.e., the transformed prevalence divided by its standard error) on study precision (defined as the inverse of the standard error). If smaller studies tend to report disproportionally higher effects, the regression intercept will deviate from zero. A statistically significant non-zero intercept ($p < 0.05$) indicates the presences of small-study effects, which may reflect publication bias. Egger's test quantifies this asymmetry and assesses whether it is statistically significant. For your information, the references we used are listed below.

1, **Sterne JAC, Egger M. Funnel plots for detecting bias in meta-analysis: Guidelines on choice of axis. J Clin Epidemiol 2001; 54: 1046-1055.** The study discussing how Funnel pots should be constructed, especially relevant when dealing with proportion or prevalence.

2, Barendregt JJ, Doi SA, Lee YY, Norman RE, Vos T. Meta-analysis of prevalence. J epidemiol community health. 2013 Nov 1;67(11):974-8. Propose the double arcsine transformation, which is essential when dealing with proportion in meta-analysis and helps when plotting funnel plot.

3, Higgins, J. P. T., Thomas, J., Chandler, J., et al. (Eds.). (2022), **Cochrane Handbook for Systematic Reviews of Interventions:** chapter 13 includes detailed discussion on reporting biases and the uses of funnel plots. It provides guidelines on interpreting funnel plot asymmetry and properly applying Egger's regression test (requiring at least 10 studies, interpretation alongside visual inspection). We welcome any feedback in case we have misunderstood your question.

Reviewers' questions or comments

3. The sensitivity analysis (p7/8) is very difficult to understand; please rephrase.

Authors' response: Thank you for your comment regarding difficulty in understanding the sensitivity analysis section. We have taken your feedback into account and made the necessary corrections in the revised manuscript. Kindly refer to page 8, lines 5-8.

Reviewers' questions or comments

4. Given the high heterogeneity observed, I understand the motivation to look for moderators. However, your sub-group analyses are not very convincing. To test whether there is time trend strikes me as a good idea. However, what you should do is include publication year (or even better: year of data collection, if available) as a moderator in the meta-analysis. You can then check if this moderator is statistically significant. This would indicate that reported violence systematically increased or decreased over the time period studied. Similarly, the comparison of countries fails to address if the level of violence differs significantly across countries. Only if that is the case should country be considered a moderator. Alternatively, you might simply drop Figures 7 and 8 and the respective text.

Authors' response: Thank you for your insightful comments regarding the subgroup analyses and the subsequent meta-regression used to investigate the sources of the observed high heterogeneity.

Given the high heterogeneity observed in the overall meta-analysis, we conducted subgroup analyses based on publication year, average sample size, and study region. Subgroup analyses by publication year and sample size did not show a notable reduction in heterogeneity within groups. However, the analysis based on study region revealed a reduction in heterogeneity in some groups.

To statistically support our subgroup findings, we conducted meta-regression analyses for all comparisons. The I^2 statistics from the subgroup analyses based on publication year and sample sizes were consistent with the meta-regression results ($p = 0.26$ for publication year and $p= 0.42$ for average sample size), suggesting these factors were not major sources of observed heterogeneity. In contrast, the subgroup analysis by study region showed a reduction in heterogeneity in some groups. However, the meta-regression result for this variable was not statistically significant ($p = 0.39$), which limits our ability to draw a definitive conclusion about

the study region as a source of heterogeneity. This inconsistency may be due to the limited number of studies within each reginal group or other unmeasured confounding factors such as differences in healthcare systems. Therefore, we acknowledge that while study region may be a potential source of heterogeneity, the current evidence is inconclusive, and the findings should be interpreted in caution. Please see page 8, lines 19-24 in the revised manuscript.

Reviewers' questions or comments

5. "Similarly, inadequate staffing was statistically associated with workplace violence" (p8): In that case the confidence interval should not include 1, but you report it as "95% CI: 0.90, 3.97" (p8). Note, if a result is statistically significant at .05 the 95% CI should exclude the value that indicates 'no effect', i.e. 1 for OR. Please address this contradiction. If staffing proves statistically non-significant, the discussion and conclusion need to change accordingly

Authors' response: Thank you for your thoughtful and thorough observation. We sincerely appreciate your comment regarding the statistical non-significance of inadequate staffing in the pooled results, as indicated by the confidence interval of the adjusted odds ratio including 1. We acknowledge your recommendation on the appropriate reporting of this non-significant association between inadequate staffing and workplace violence. We apologize for the mistake we made. In response, we have revised the discussion and conclusion sections accordingly to reflect this more accurately. Please see page 11, lines 1-8.

Reviewers' questions or comments

6. Table 2: It is essential that you indicate for each study, what measure of workplace violence was used (or that relevant information is missing). If we don't know what the studies asked, we don't know what the overall prevalence means. If the studies assessed the prevalence of workplace violence in different ways, this might have contributed to the large observed heterogeneity.

Authors' response: Thank you for four feedback regarding the missing information on the characteristics of the included studies. We have taken your feedback into account and added the necessary information on the characteristics of the included studies as supplemental information. Kindly refer page 7, lines 18-20 and **supplemental 3 file**.

Reviewers' questions or comments

A minor point: It should be "Likewise" instead of "Like ways" on p10.

Authors' response: Thank you for your thorough review of the manuscript. We have made the necessary corrections in the revised manuscript. Please see page 10, line 18 in the revised manuscript.
