# Supplementary material for: Magnitude of workplace violence and associated factors among healthcare professionals in East Africa: A systematic review and meta-analysis
Source: PLoS One. 2025 Sep 19;20(9):e0332415. doi: 10.1371/journal.pone.0332415 (PMC12448341; doi:10.1371/journal.pone.0332415)
Supplement: Attachment — Submitted filename: Response_to_reviewers_auresp_3.pdf [file pone.0332415.s008.pdf]

Point by point response for editor and reviewers

Journal Name: PLOS ONE, Manuscript Number: PONE-D-24-46926R2

Title: Magnitude of workplace violence and associated factors among healthcare professionals in East Africa: A Systematic Review and Meta-Analysis.

To editor

We sincerely thank the editor for granting us the opportunity to resubmit our revised manuscript. We greatly appreciate the valuable comments, thoughtful guidance, and the time and effort invested in helping us improve the quality of our work.

Point by point response to Reviewer 2

Dear reviewer, we are truly grateful for the time and effort you dedicated to reviewing our manuscript. Your insightful comments and constructive suggestions have been invaluable in guiding our revisions and have significantly contributed to improving the clarity, coherence, and overall quality of the paper. We sincerely appreciate your thoughtful feedback and the role it has played in strengthening our work

Reviewer #2: Thank you for the opportunity to review the new version of this manuscript. I have two minor comments from this new version and the responses provided to the reviewers

Reviewers' questions or comments

1,The manuscript says that "this review included all quantitative and mixed-methods observational studies". This is an inclusion criterion. Later, explaining the included and excluded articles, the manuscript says that 13 studies were excluded because the design did not align with inclusion criteria. However, I understand that those 13 studies reported the desired outcome, which is a prevalence, and thus I also understand that those 13 studies were quantitative. Of couse, I do not know which studies were excluded for this reason, but I can not help but wonder how were those studies performed to have the desired prevalence and yet not being included.

Authors' response: Thank you for your comments regarding the study selection process, specifically the exclusion of the 13 studies.

The primary and secondary outcomes of interest in this study are the prevalence of workplace violence and its associated factors, respectively. Accordingly, we included studies that report either prevalence and/or associated factors of workplace violence. Although systematic reviews, meta-analyses, clinical trials, case studies, and qualitative studies can report these outcomes, they were excluded due to differences in study design. This explains the exclusion of the 13 studies. However, reporting the outcomes of interest does not necessarily imply that the studies exclusively quantitative, as qualitative studies can also report associated factors for violence. For example, see the study, ***"Workplace Violence against Nurses in Uganda's Public Hospitals: A Phenomenological Study"*** **(available at: Makerere University Repository)**. Please use the following link to access it. https://makir.mak.ac.ug/handle/10570/14186. The most appropriate exclusion criterion for this study is the study design, rather than the study area, study population,

or outcome of interest. We welcome any suggestion for further improving the clarity of this expression.

Reviewers' questions or comments

2, I agree with a comment formerly made by another reviewer that meta-regression using year as the independent variable would be a good idea. First, perhaps re-ordering the studies in Figure 2 by year would allow us to see possible time trends in the prevalence of workplace agressions; I must admit I do not see how studies in Figure 2 are currently ordered and perhaps a different order could be more helpful, but I would not be very insistive on this. Second, and more importantly, the authors divided the studies in two groups: before 2022 and after 2022, but there is still much heterogeneity. I would suggest treating the year as a quantitative variable in the meta-regression, instead of dichotomizing it.

Authors' response: Thank you for your valuable comments regarding the reordering of figure 2 and the use of year as moderator in the meta-regression analysis to explore the source of variability among the included primary articles.

1, Figure 2 presents the pooled prevalence of workplace violence, the individual study estimates, heterogeneity, and the study weights. In fact, we did not consider the publication year, the alphabetic order of the first author's name, or any other characteristics when constructing the forest plot of the pooled prevalence of workplace violence. For your information the current order is based on the study region. There was no specific intention behind this ordering—it simply reflects the sequence of data collection. Arranging studies chronologically by year of publication could allow readers to visually perceive weather prevalence is increasing or decreasing over time; however, this would only provide a rough visual impression, not a formal statistical test of trend. To determine whether the prevalence of workplace violence significantly changes over time, a meta-regression analysis would be required. We are willing to adjust the ordering of figure 2 if deemed necessary.

2, Given the heterogeneity among the included articles, we employed several methods to explore its potential sources. One approach was subgroup analysis based on year of publication, however, this did not reduce heterogeneity within the groups. This may be due to the small number of studies in each group or other unmeasured factors. We also conducted a meta-

regression using year of publication as moderator, but the result was not statistically significant ($p = 0.26$). For details please see page 8, lines 10 - 24.

Point by point response to Reviewer 3

Dear reviewer, we are sincerely grateful for the time and effort you dedicated to reviewing our manuscript. Your insightful comments and constructive suggestions have been invaluable in guiding our revisions and have greatly contributed to enhancing the clarity, coherence, and overall quality of the paper. We deeply appreciate your thoughtful feedback and the role it has played in strengthening our work

Reviewer #3: The current version of the paper reads better and has improved in quality once again. Conducting a meta-analysis is a time consuming and hard work - I am very aware of this.
There are a few minor improvements that I recommend below.

The authors have argued well why they had to exclude many studies. The explanations for the three studies I mentioned have shown me this in an exemplary manner.

I would like to thank the authors for their great patience in responding to my objections with the relevant explanations and apologise once again for my mistrust.
From the studies included in the meta-analysis and their composition in terms of the countries in which they were conducted, I conclude that there is a significant lack of high-quality studies on the topic of WPV in the healthcare sector in most East African countries. This could be a conclusion from your study.

Reviewers' questions or comments

Points for improvement:
- You write: "20% to 38% of healthcare workers have experienced physical violence at some point during their careers, compared to employees in other sectors."
This concerns this sentence in the abstract and a similar sentence in the introduction.
I am missing the comparison. I would add some values of WPV from other areas here.

Authors' response: Thank you for your comment regarding the need to add a numeric value for comparison. We have taken your feedback into account and incorporated the numeric value accordingly. Please see page 3, line 11.

Reviewers' questions or comments

- In the abstract, you list the associated factors three times in a row, once in the same sentence (with and without AOR values) and then again in the conclusion.

I recommend rephrasing the sentence and the conclusion.

Authors' response: Thank you for your insightful comments regarding the unnecessary repetition of similar expressions about associated factors. We have accepted your constructive feedback and made the necessary corrections accordingly. Kindly refer to page 2 of revised manuscript.

Reviewers' questions or comments

- Result section: 'An extensive search was conducted, and a total of 1,243 studies were retrieved from databases such as Google'

better: 'An extensive search was conducted, and a total of 1,243 studies were retrieved from the databases Google, ...'

Authors' response: Thank you for your suggestion regarding improving the sentence structure. We have taken your comment into account and incorporated it into the revised manuscript. Please see page 7, line 4.

Reviewers' questions or comments

- Publication bias: 'The p-value from Egger's regression test ($p < 0.03$), along with the asymmetric distribution of the included primary studies on the funnel plot (Fig 3a), and suggests the presence of publication bias.'

-> 'The p-value from Egger's regression test ($p < 0.03$), along with the asymmetric distribution of the included primary studies on the funnel plot (Fig 3a), suggests the presence of publication bias.'

Authors' response: Thank you for your valuable comment regarding sentence clarity. We have corrected in the revised manuscript. Kindly refer to page 7, line 28.
